# Measurement of Intervertebral Motion Using Quantitative Fluoroscopy: Report of an International Forum and Proposal for Use in the Assessment of Degenerative Disc Disease in the Lumbar Spine

**DOI:** 10.1155/2012/802350

**Published:** 2012-05-16

**Authors:** Alan C. Breen, Deydre S. Teyhen, Fiona E. Mellor, Alexander C. Breen, Kris W. N. Wong, Adam Deitz

**Affiliations:** ^1^Institute for Musculoskeletal Research and Clinical Implementation, Anglo-European College of Chiropractic, 13-15 Parkwood Road, Bournemouth BH5 2DF, UK; ^2^Fort Sam Houston, US Army-Baylor University, San Antonio, TX 78234, USA; ^3^Faculty of Science & Technology, Technological and Higher Education Institute of Hong Kong, Hong Kong; ^4^Ortho Kinematics Inc., 1704 Bee Caves Road, Building 3, Suite 315, Austin, TX 78746, USA

## Abstract

Quantitative fluoroscopy (QF) is an emerging technology for measuring intervertebral motion patterns to investigate problem back pain and degenerative disc disease. This International Forum was a networking event of three research groups (UK, US, Hong Kong), over three days in San Francisco in August 2009. Its aim was to reach a consensus on how best to record, analyse, and communicate QF information for research and clinical purposes. The Forum recommended that images should be acquired during regular trunk motion that is controlled for velocity and range, in order to minimise externally imposed variability as well as to correlate intervertebral motion with trunk motion. This should be done in both the recumbent passive and weight bearing active patient configurations. The main recommended outputs from QF were the true ranges of intervertebral rotation and translation, neutral zone laxity and the consistency of shape of the motion patterns. The main clinical research priority should initially be to investigate the possibility of mechanical subgroups of patients with chronic, nonspecific low back pain by comparing their intervertebral motion patterns with those of matched healthy controls.

## 1. Introduction

The need to be able to measure intervertebral motion in the diagnosis of problem back pain has been recognised for over a century. Attempts began with plain X-ray studies [1–5] and were followed by cineradiography [6–10], videofluoroscopy [11–16], roentgen stereophotogrammetry [17, 18], and magnetic resonance imaging [19, 20]. All have been found impractical for routine clinical use for a variety of reasons, ranging from poor image quality to low computing power, poor reliability and accuracy, laboriousness of multiple image registrations, X-ray dosage, invasiveness, cost and problems with sequential image acquisition. Until the emergence of quantitative fluoroscopy technologies, the standard approach to evaluating the mechanics of intervertebral linkages *in vivo *has remained a pair of plain radiographs taken at the end of bending range [21].

Quantitative fluoroscopy (QF) is an objective assessment of the spine in motion using fluoroscopy (moving video X-rays) and automated computer processing algorithms which calculate intersegmental kinematic parameters throughout the motion. It overcomes the above obstacles by automatically processing low-dose digital fluoroscopic image sequences from live subjects in motion [16, 22–25]. The method uses modern conventional image intensifiers and requires little specialist knowledge to operate. However, differences between the techniques of different research groups have made comparison of results difficult; therefore a consensus is needed if it is to benefit patients.

By 2008, three independent teams from across the world had published methods and results from their individual studies. Their varying approaches to acquisition, analysis, and interpretation meant that combining or comparing data was impractical and a more standardised approach, building on the strengths of the different methods was desirable. In August 2009, with support from the British Council in the form or a grant under the International Networking for Young Scientists Scheme, these three teams met in San Francisco for the First International Forum on Quantitative Fluoroscopy of the Lumbar Spine. This International Forum was a networking event of the three research groups (UK, US, Hong Kong), over three days. Its aim was to reach a consensus on how best to record, analyse, and communicate QF information for research and clinical purposes.

## 2. Materials and Methods

Three research teams led by Professor Alan Breen (AB) (UK), Dr Deidre Teyhen (DT) (US), and Dr Kris Wong (KW) (Hong Kong) met over three days to attempt to reach consensus on a proposal for optimal QF methodology for clinical and research studies. The Forum was also attended by representatives from the medical devices company Ortho Kinematics Inc., also of the US. After discussion on the rationale for quantitative fluoroscopy, the teams considered 4 subject areas: (1) choice of intervertebral motion measurement, (2) image sequence acquisition protocols, (3) image analysis methods, (4) future research priorities. Each team, in turn, described its methodology, followed by group discussions on a consensus in each area.

All sessions were recorded and transcribed to note form by FM. Two drafts of the proceedings were compiled by AB and circulated for comment and amendment. Further drafts of some sections were written by DT and FM. A final compressed version for publication was edited by AB with input from all groups. Updates on reliability and accuracy were obtained from FDA studies in 2011 and for radiation dosage from the masters' degree dissertation of one author (ACB).

## 3. Results and Discussion

### 3.1. Choice of Intervertebral Motion Measurement

There is a range of options for acquiring intervertebral motion data for measurement, for example, in the coronal or sagittal plane (the transverse plain not being assessable); in lying, sitting, or standing orientations; using free or controlled bending protocols and using various methods for patient stabilisation. There are also options for what to measure to best inform clinical decisions. These traditionally include overall angular rotation and translational range of intervertebral motion (IV-ROM), the position(s) of the instantaneous axis of rotation (IAR) [26, 27], and laxity in the form of the size of the Neutral Zone [28]. QF acquires continuous motion data, offering possibilities to measure all of these, plus others, such as the proportions of lumbar motion shared by the various levels [29], “phase lag” (the tendency for different levels to commence or end at different points in the trunk motion sequence) [30] and the measurement of disc height [31]. Other important choices include those of vertebral landmarks and their use to calculate these. The technique as a whole also depends on the minimisation of radiation dosage, the reduction of movement blurring and the avoidance of out-of plane image distortion.

The Forum agreed on the following 7 priorities for measurement by QF:

1. Range of intervertebral rotation.
2. Range of intervertebral translation.
3. Directional coherence.
4. Motion commencement sequence.
5. Neutral zone laxity.
6. Instantaneous axis of rotation (IAR).
7. Disc height.

### 3.2. Image Sequence Acquisition Protocols

The US method [22] assessed lumbar flexion and extension in the upright posture. The subjects move through their full range of motion and are instructed to slowly bend forward and return to the upright posture in about 4-5 seconds. This pace was selected based on patient comfort and that faster movements could result in blurring of the images. Subjects complete four cycles of flexion, and extension, with the third cycle captured for analysis. To help maintain the lumbar spine within the field of view and minimize hip and knee flexion a stabilization device that included a climbing harness and belts was used to stabilize the patient. The Hong Kong method [32] also acquired flexion-extension images in the standing position with an electrogoniometer strapped to the back [15, 23, 32] and the pelvis unconstrained. Subjects voluntarily extend and flex maximally and then return to neutral. The intensifier was made to follow and keep the vertebrae of interest in the middle of the field. This may result in movement blurring.

**Figure 1:**
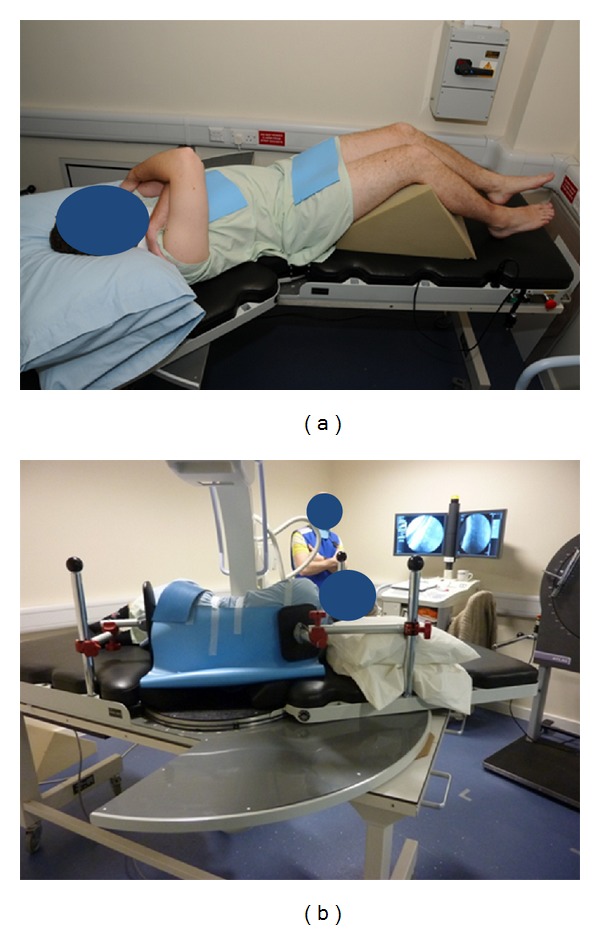
(a) Passive recumbent supine right lateral flexion acquisition. (b) Passive recumbent flexion acquisition.

**Figure 2:**
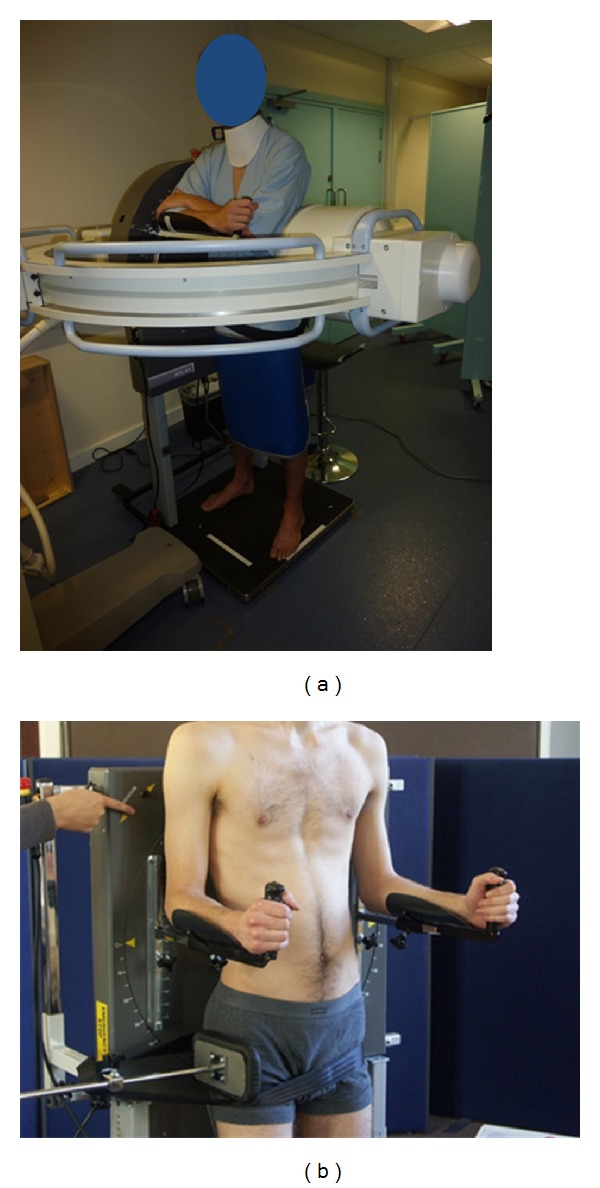
(a) Weight-bearing flexion-extension image acquisition following an upright motion frame. (b) Weight-bearing side-bending acquisition: position of motion frame.

The UK method screened subjects in either passive, controlled recumbent motion on a specially designed motion table (Figures 1(a) and 1(b)) [24] or standing against a special motion frame (Figures 2(a) and 2(b)) (Atlas Clinical Ltd.). This method measured both flexion and extension, used lead masking to reduce intensifier flare during motion, and controlled for rate and range. This was conventionally 40 degrees in each outward direction over 10–15 seconds for each direction.

Consensus: The Forum agreed that imaging procedures should include both the standing and lying patient orientations and both the coronal and sagittal planes, with the sacrum stabilised during weight bearing investigations with the patient following an upright motion frame to control the rate and range of trunk motion (Figures 2(a) and 2(b)). No restraint is needed for lying acquisitions (Figure 1) where knee support in the supine position and antiroll pads in the lateral recumbent position can provide adequate stabilisation.

All image capture should be preceded by “warmup” motion (without fluoroscopic screening). The simultaneous recording of trunk motion is inherent in all three methods as continuous global motion data are needed to make comparative calculations with kinematic measurement. The UK method imposed preset global motion on the trunk, whereas the Hong Kong method used surface goniometry which may be unreliable [33] and the US method measured from the vertebral images over a short section of the lumbar spine. It was decided to recommend the UK method; however, this may not challenge all segments in very flexible subjects. Therefore, it was also recommended that free, end-of-range and neutral fluorograbs are obtained to check that any fixed segments have been adequately challenged before accepting a finding of immobility.

It was agreed that the range and velocity of trunk motion should be standardized, and all image acquisition should start from the neutral position. This reduces the global range variability making possible the collection of normative intervertebral motion information and allowing follow-up studies to have standardised comparators. A neutral position start also ensures that Neutral Zone information can be obtained. However, it is recognised that, in the lying positions the flexing of the patient's knee and hips means that the lumbo-sacral spine is also slightly flexed.

It was also recommended that the standardised range for recumbent motion is 40 degrees in left, right, and flexion directions for both standing and lying investigations, with the exception of 20 degrees of extension and 60 degrees of flexion for flexion-extension motion in weight bearing, which takes account of the natural lumbar lordosis in the erect postures. In order to avoid “aliasing” or movement blurring if acquisition is too slow or too fast, it was recommended that each motion direction duration is of 8–12 seconds, with ramp-up, ramp-down, and motion reversal intervals of 0.5–1 seconds to avoid lost image registration at the beginning and patient “wobble” at the end of ranges.

A single unidirectional fluorosequence should involve around 20 seconds of exposure, including positioning and use factors between 70–90 kVp and 50–70 mA. A whole examination involving flexion-extension and left-right lateral flexion should give an average effective dose of between 0.80 and 1.5 mSv. (This can be compared to 1.3 mSv which is the reported average dosage for an AP and lateral single plain radiographic series of the lumbar spine [34, 35]).

The US method captures images at 30 fps using a digital frame grabber and the UK method at 15 fps taken directly from a digital fluoroscope. It was recommended that at least 8-bit images acquired at 15 fps over 6–20 seconds of motion would be acceptable and that image acquisition speed should be not less than 12.5 fps and digital image bit-depth and pixel densities not less than 8-bit and 512 × 512, respectively.

### 3.3. Image Analysis Methods

QF image sequences can provide several hundred images per examination. To use these for kinematic measurement therefore requires automated methods. The steps involved are image registration, image tracking, recording of serial intervertebral spatial relationships throughout the motion, transformation of these spatial relationships as data outputs, and the summarisation of these outputs into graphic or numerical form for interpretation.

**Figure 3:**
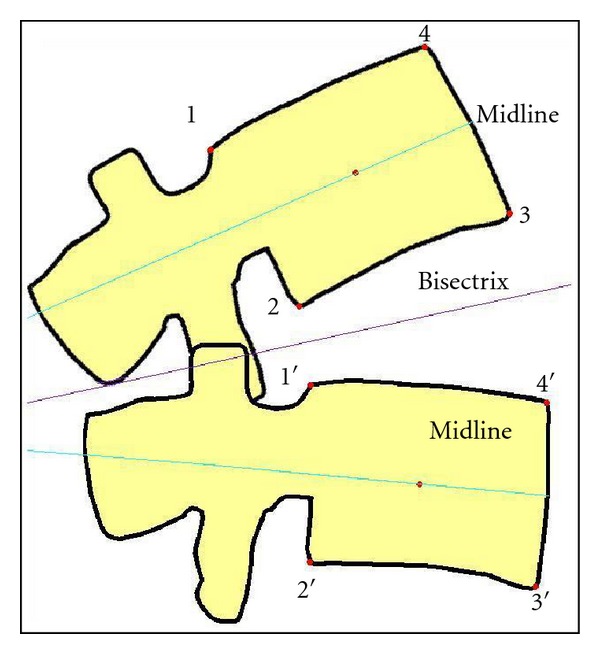
Frobin et al.'s [36] method for registering the positions of vertebrae.

In the US method, images were enhanced to help detect the borders of the vertebral bodies from the surrounding soft tissue using digital filters (Image Pro Plus software, MediaCybernetics, Silver Springs, MD). Images were then imported to MATLAB (The Math Works, Natick, MA) for vertebral body detection and kinematic analysis. Vertebral body detection consisted of manually defining the vertebral body corners and specific midpoint locations using a modified technique originally developed by Frobin et al. [36] (Figure 3). Following this, computer algorithms were used to verify these corner locations and calculate the specific midpoint locations. Four iterations of the vertebral corner selection process were used to enhance reliability. Once these locations were determined for each frame (approximately 200 frames per flexion-extension cycle), the key points to detect the vertebral body were smoothed across frames using a fourth-order Butterworth filter to minimize error.

In the Hong Kong, method the 4 corners of the vertebral body images are marked. This is referred to as the “active contour method,” or “Snake.” The active contour program fits a template, and an image processing program then fits this to the edges of subsequent vertebral images by learning the outline and predicting the position of the next template in the sequence. This is thought to be highly reliable over the same images because the active contour method always finds the same edges. This is true for measuring rotation, but translational motion is error-prone because, unlike the Frobin et al. method, this method does not compensate for image distortion. From acquisition, intervertebral motion is measured for every degree of trunk motion from 20 degrees extension to 40 degrees flexion as measured from L1 to S1. This method is not significantly inhibited by the presence of bowel gas or intensifier flare.

**Figure 4:**
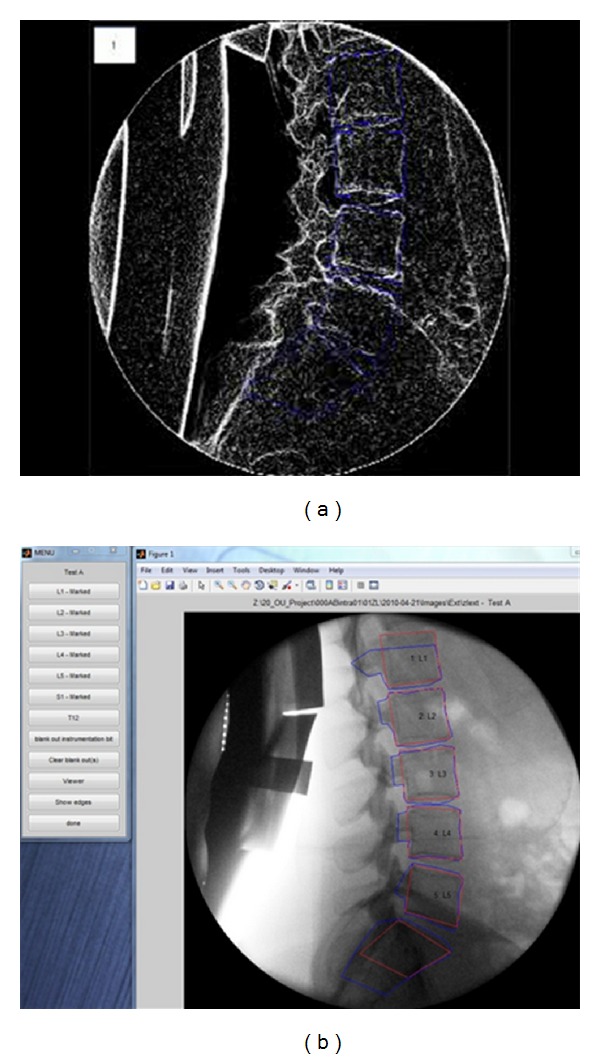
(a) Lateral view of lumbar spine image with enhancement. (b) User interface output showing lateral view of lumbar spine image with tracking and reference templates.

In the UK method, images are also enhanced (Figure 4(a)). The resultant images are marked by placing cursor lines around each vertebral body five times (tracking templates). These are registered from frame-to-frame automatically throughout the sequence using cross-correlations and a rolling average over each 2 images as the sequence progresses to reduce noise. During marking, additional templates (reference templates) are placed using only the four body corners and are linked to the tracking templates as coordinates in order to verify tracking and to obtain coordinates for calculating translation, disc height, and IAR using the Frobin et al. method [36, 37]. (Rotation is calculated from the vertebral tracking templates individually).

Areas of implanted metal within the vertebral images can be removed by marking around them and subtracting out the enclosed area. The ability of the templates to track all images is checked both by viewing the overlay of the vertebral motion graphs and the adherence of the templates to the vertebrae during video playback. For intervertebral rotations, the 5 trackings for each vertebra are subtracted from those adjacent to them for each combination of vertebrae and vertebral tracking to give 25 intervertebral angle sequences per pair. Mean and median values of these 25 are very similar and either can be used to display rotational results. Failed trackings may be remedied by remarking vertebrae.

Consensus: Although each research team addressed QF differently, a combination of best practices across the techniques has the possibility of improving the technology. This may be achieved by using the US method for more reliably locating corners in the initial images, followed by the Hong Kong method for fitting the templates to the vertebrae and then the UK method for tracking them. It should then be possible to combine the advantages of automated tracking with more precise template fitting to obtain more reliable results with less operator interaction. The Hong Kong method for tracking could also be used as an alternative in individual patients.

It would also be useful to try to test these multiple methods with the same patient. This would involve first, corner marking, then corner detection, then marking reference templates based on the Snake, then placing these reference templates in the Snake for one of the five tests and tracking the rest with cross-correlations. The cross-correlation method is based on the rigid-body assumption, and this should give better results than the Snake method (which changes shape during tracking) for calculating translation, disc height and IAR, whereas using the Snake method for one of the trackings should give better results for rotations.

This should also accommodate the need to blank out metallic implants; however, the Snake method is as yet untried for A-P images and may also not track S1 in the lateral projection. To optimise image analysis using a combination of these methods will require optimal image acquisition and an understanding of the effects of body type on the different image processing technique combinations.

### 3.4. Indices of Motion

All groups had used QF to determine rotational IV-ROM, but it was recognised that various geometric transformations of the data would provide access to many more kinematic parameters.

**Figure 5:**
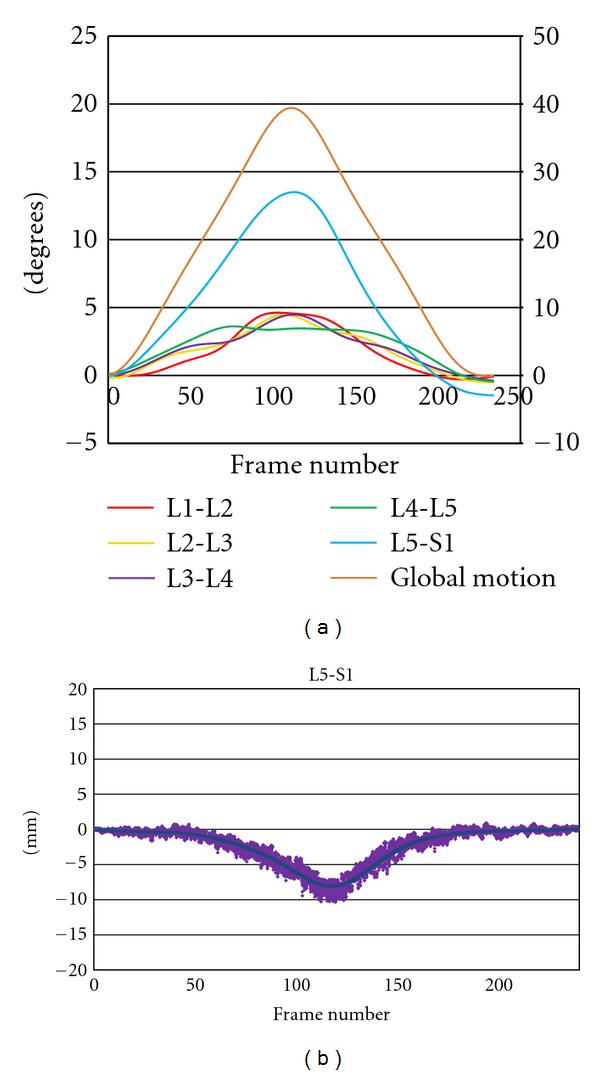
(a) Continuous intervertebral angles for 5 levels (left *y*-axis) and global (trunk) motion (right *y*-axis) in a patient with unstable L5-S1 spondylolytic spondylolisthesis. (Passive recumbent extension motion, note excessive motion at L5-S1, with irregular motion at L3-4 and L4-5. Maximum L4-5 range attained before maximum global motion range). (b) Translational motion path at L5-S1 extension in the same image sequence as in 5(a). (Solid line is mean translation and shaded area is all data). Note translational range of 8 mm.

It was decided to prioritise rotational and translational range, regularity, symmetry, laxity, and IAR location in recumbent passive and active weight-bearing configurations as useful measurements in people with chronic, nonspecific low back pain. Continuous rotational and translational range data provides the measurement of maximum range, wherever it is attained during trunk motion, while enabling, by data extrapolation, the display and measurement of phase lag, motion sharing, regularity, and laxity (Figures 5(a) and 5(b)).

**Figure 6:**
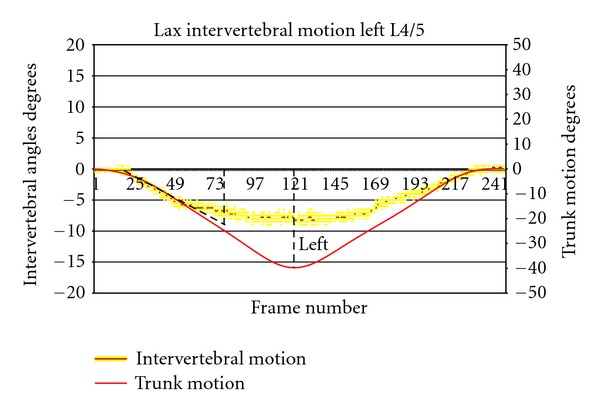
Example of use of intervertebral versus trunk motion graphs for the calculation of laxity by ratio of their slopes in the first 10 degrees of global motion (slope of global motion = −0.536).

The measurement of Neutral Zone laxity has been subject to some preliminary testing using recumbent lateral bending studies [25]. The ratio of the slopes of intervertebral and global motion is measured in the accompanying 10 degrees of trunk motion. The higher the ratio of intervertebral motion slope to global motion slope, the less restraint is acting in the Neutral Zone (Figure 6).

**Figure 7:**
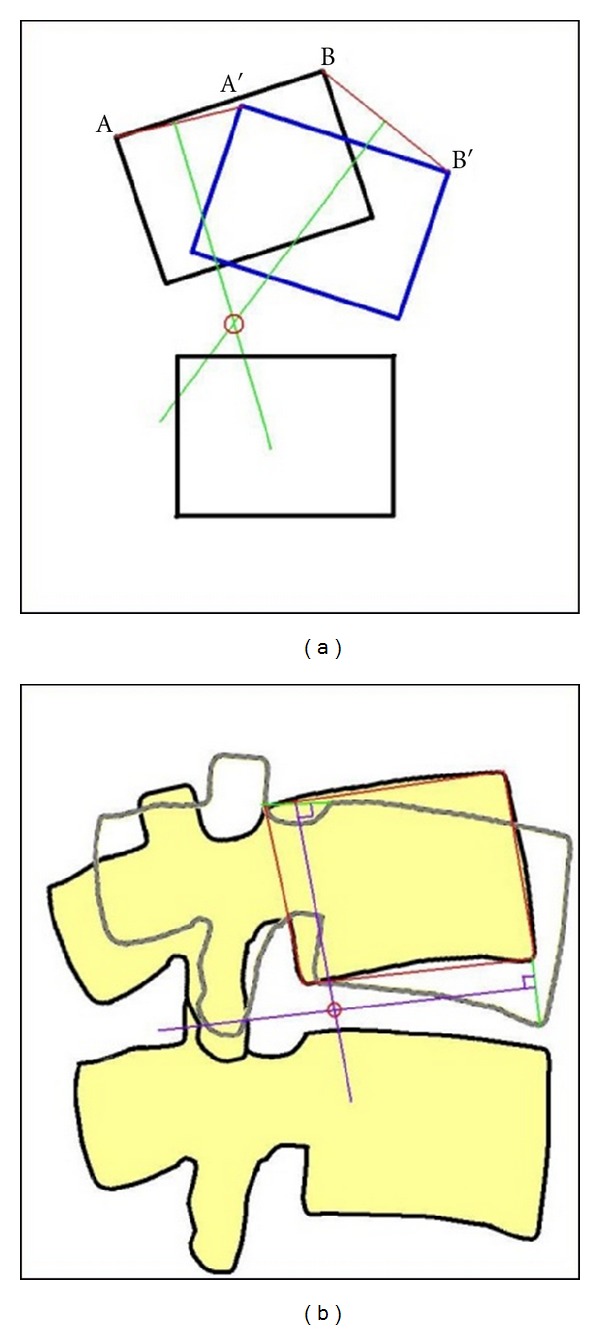
Illustration of geometric determination of IAR: (a) on a simple block diagram, (b) on vertebral body images. The IAR is located in the posterior half of the disc space.

**Figure 8:**
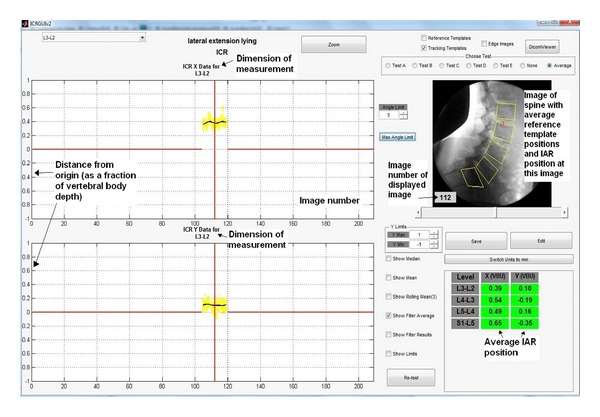
User interface image showing graphical and numerical output of overall IAR position and as the location of this position on the image.

IAR is also computed from the same reference template information as the other motion parameters (Figures 7(a) and 7(b)). This can be displayed as *x*-*y* coordinates, equivalent mm from a nominated anatomical landmark, graphically on the user interface or as a location on the image (Figure 8). Multiple IARs can also be computed serially and displayed as moving or accumulating points on a video sequence of the images.

### 3.5. Repeatability and Accuracy

All groups at some time have also reported on the reliability and/or accuracy of QF for intervertebral range measurement. The most recent accuracy calculations come from a 2011 FDA study (Ortho Kinematics 2011) which used 60 image sets from two *in vitro* calibration models made of human vertebrae. The QF images were distorted by rotating half of them 10 degrees out of plane, and all were degraded by interposing animal soft tissue. The results for intervertebral rotation report an error of less than 0.70 degrees for rotational measurement and less than 2.60% of vertebral body depth for translation (<0.91 mm for a standard vertebra of 35 mm depth) (Table 1). 

The repeatability part of this study compared three measurement methods: QF, digitisation of X-rays at maximum voluntary bending angles (MVBA), and measurement of X-rays at MVBA by ruler and protractor. Intervertebral rotation and translation were recorded in 63 patients' image sequences by 3 trained observers. The mean RMS errors for all patients and intervertebral levels are shown in Table 2, reflecting repeatability errors of less than 1.30 degrees and 1.92% of vertebral body depth (0.7 mm) for QF compared to substantially larger errors for the other two methods.

### 3.6. Radiation Dose

QF uses low-exposure durations compared to what is traditionally expected of fluoroscopy. This and improved image intensifier technologies keep the radiation dosages low. The average dose across 53 subjects who underwent QF examination in the UK in 2011 (passive motion flexion-extension and right and left lateral bending) was 0.89 mSv, with a standard deviation of 0.25 mSv. This is equivalent to approximately 22 weeks of UK average background radiation [34] (where the UK average is 2.2 mSv per year). As a comparison, the typical dose received during an X-ray examination of the hip is 0.3 mSv, equivalent to 7 weeks background radiation or additional lifetime risk of 1 in 67,000 fatal cancer per examination. An X-ray examination of the thoracic spine is 0.7 mSv (4-month background radiation or 1 in 30,000 lifetime risk of fatal cancer per examination) and an examination of the lumbar spine is 1.3 mSv (7-month background radiation, 1 in 15,000 lifetime risk).

### 3.7. Future Research Priorities

Multiple authors have researched fluoroscopy as a method for measuring intervertebral motion *in vivo* [6, 7, 9, 10, 12, 14, 15, 38–48], but it has only recently been developed as a diagnostic technology. The reasons for this include a lack of a suitable methods for standardising patient motion, assuring adequate image quality, achieving frame to frame image registration and obtaining adequate computer online storage and processor speed to handle the required volume of image data. However, once these began to appear, QF became a viable method and its reliability, validity, X-ray dosage [24], and clinical utility [25, 29, 32, 49] began to be investigated.

The benefits to patients from QF will be principally in the conservative care arena, where most people remain, but also in the world of spinal surgery, where the more severe cases are often found and where many implantable devices that are intended to influence intervertebral motion require evaluation.

The Forum identified, as a priority for future QF research, the investigation of mechanical subgroups within chronic nonspecific low back pain and disc degeneration. However, large subject populations are needed to establish subgroups. This is not only because the main beneficiaries will be the minority of patients who have chronic pain, but also because the consequences of ligament subfailure involve combinations of abnormalities [50] of the passive, active, and control systems of the spine [28, 51–53]. In conservative treatment, for example, strength alone may not be enough to rehabilitate if motor control is not improved. It is therefore necessary to find methodologies that will disaggregate these for clinical purposes. This anticipates combining QF with other technologies, such as electromyography and algometry to investigate more thoroughly these patient subgroups.

One promising entry point into these lines of investigation of data may lie in studies of the lumbar multifidus muscle and the changes in its function and structure that occur in chronic back pain [54–56]. We also need to understand the role of other trunk musculature, notably the transversus abdominus in these syndromes [54]. Using QF and other technologies in combination, it may be possible to discover when and to what extent chronic back pain may be associated with recordable abnormalities in the passive, active, and control systems as separate entities.

It is also recognised that psychosocial factors can play a part in prognosis [57, 58] and patient populations in QF subgrouping studies should take account of the extent to which these are present. For example, in terms of intervertebral function, the role of fear-avoidance behaviours [59] is unknown.

## 4. Conclusion

People with chronic, nonspecific low back pain are likely to be a very heterogeneous group. However, an objective diagnostic test that could help guide its management would be valuable for individual patients and society as a whole. These benefits would lie in being able to better predict who will benefit from spinal manipulation, exercises, and flexible stabilisation surgery. It may also predict who will return to work, who will need to leave their jobs, and who will become dependent on social support. Previous research has identified a number of weak to moderate predictors of these outcomes, but none have been able to objectively assess an intervertebral site that is suspected of being mechanically involved. In the future, QF technology may be used to determine which patients with chronic nonspecific back pain had a mechanical basis for it.

It will also be necessary to know the intrasubject variation in pain-free subjects over a treatment period. These intrasubject reliability studies in control subjects will be necessary to ascertain the smallest change over time that could be attributed to a treatment intervention. Clinicians from both the surgical and conservative care will then be able to investigate the role of mechanics in patient outcomes.

## Figures and Tables

**Table 1:** Accuracy: combined results from two calibration models for four bending modes.

	RMS error			
Study	Flexion	Extension	Left	Right
Rotation (degrees)	0.69	0.57	0.1	0.22
Translation (% body depth)	2.44	2.59	N/A	N/A

**Table 2:** Observer repeatability for three measurement methods (mean RMS).

	Intraobserver errors			Interobserver errors		
Study	QF	MVBA	Ruler	QF	MVBA	Ruler
Rotation (degrees)	0.77	2.66	4.22	1.26	3.14	4.50
Translation (% body depth)	1.19	3.83	5.83	1.92	4.35	6.61
